# Electronic confinement induced quantum dot behavior in magic-angle twisted bilayer graphene†

**DOI:** 10.1039/d4nr02824d

**Published:** 2024-12-23

**Authors:** Bhaskar Ghawri, Pablo Bastante, Kenji Watanabe, Takashi Taniguchi, Michel Calame, Mickael L. Perrin, Jian Zhang

**Affiliations:** a Transport at Nanoscale Interfaces Laboratory, Empa, Swiss Federal Laboratories for Materials Science and Technology 8600 Dübendorf Switzerland michel.calame@empa.ch mickael.perrin@empa.ch; b Departamento de Física de la Materia Condensada, Universidad Autónoma de Madrid 28049 Madrid Spain; c Research Center for Electronic and Optical Materials, National Institute for Materials Science 1-1 Namiki Tsukuba 305-0044 Japan; d Research Center for Materials Nanoarchitectonics, National Institute for Materials Science 1-1 Namiki Tsukuba 305-0044 Japan; e Department of Physics, University of Basel 4056 Basel Switzerland; f Swiss Nanoscience Institute, University of Basel 4056 Basel Switzerland; g Department of Information Technology and Electrical Engineering, ETH Zurich 8092 Zurich Switzerland; h Quantum Center, ETH Zürich 8093 Zürich Switzerland; i Max Planck Institute of Microstructure Physics Weinberg 2 Halle 06120 Germany jian.zhang@mpi-halle.mpg.de

## Abstract

Magic-angle twisted bilayer graphene (TBLG) has emerged as a versatile platform to explore correlated electron phases driven primarily by low-energy flat bands in moiré superlattices. While techniques for controlling the twist angle between graphene layers have spurred rapid experimental progress, understanding the effects of doping inhomogeneity on electronic transport in correlated electron systems remains challenging. In this work, we investigate the interplay of confinement and doping inhomogeneity on the electrical transport properties of TBLG by leveraging device dimensions and twist angles. We show that reducing device dimensions can magnify disorder potentials caused by doping inhomogeneity, resulting in pronounced carrier confinement. This phenomenon is evident in charge transport measurements, where the Coulomb blockade effect is observed. Temperature-dependent measurements reveal a large variation in the activation gap across the device. These findings highlight the critical role of doping inhomogeneity in TBLG and its significant impact on the transport properties of the system.

## Abstract 2

Reducing the dimensions of magic-angle twisted bilayer graphene devices can amplify disorder potentials caused by doping inhomogeneity, leading to enhanced carrier confinement.

## Graphical abstract

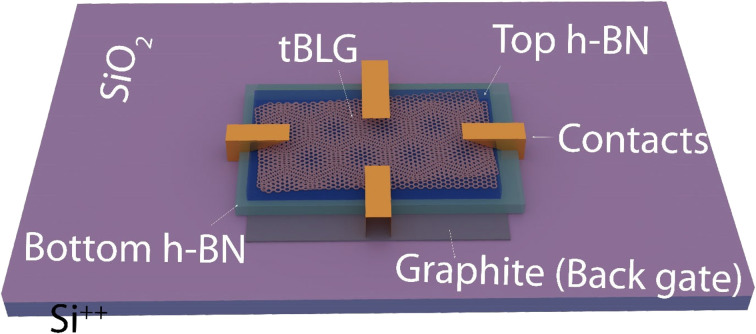

## I. Introduction

Twisting two layers of graphene creates a moiré superlattice leading to a strongly tunable, twist angle-dependent band structure.^1,2^ Near the so-called magic angle of *θ*_m_ ≈ 1.1°, the bands become almost dispersionless, and twisted bilayer graphene (TBLG) exhibits remarkable phenomena such as Mott insulator behavior,^3,4^ superconductivity,^4,5^ ferromagnetism,^6–8^ Chern insulator behaviour, non-Fermi liquid behaviour,^9–11^ and other correlated electron states.^12–14^ These phenomena arise from the intricate interplay between electron–electron interactions, lattice structure, and non-trivial band topology.^15^ However, TBLG exhibits two seemingly contradictory characters at similar twist angles—localization and quantum-dot-like behavior, as observed in scanning tunneling microscopy (STM) experiments,^16,17^ alongside delocalization or superconductivity in transport experiments.^4,5,18^ This duality has recently been explained by modeling TBLG using the topological heavy fermion problem.^19,20^

Despite significant advancements in improving the device quality and understanding the origins of these many-body phases, a large sample-to-sample variation still remains a major challenge in this field.^13,14^ These variations can arise either from differences in the twist angles across the sample,^21–23^ or from inhomogeneities in the local doping of the sample.^24^ Although STM measurements have provided insights into carrier confinement in magic-angle TBLG,^24^ a systematic study investigating the effect of quantum confinement and doping inhomogeneity remains unexplored in charge transport measurements, primarily because most of the studied devices are too large to observe the signatures of doping inhomogeneity in such measurements. In this work, we have performed electric transport measurements in TBLG (*θ* ∼1.19–1.65°) patterned in a rectangular shape that is small enough for confinement effects and local doping to significantly affect the charge transport properties. In particular, we observed signatures suggesting that charge carriers are confined in conducting regions separated by insulating regions, resulting in quantum dot behavior near the edge of the flat bands. Furthermore, this effect becomes more pronounced as the twist angle is tuned closer to the magic angle.

## II. Results and discussion

**Fig. 1:**
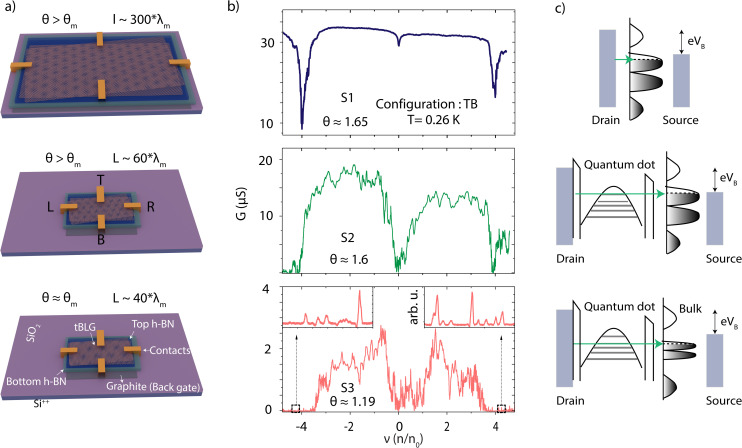
Device structure and electric characterization. (a) Schematics showing the three different type of devices probed in the study. The constituent layers, electrical contacts, and the gate assembly are marked. The graphite layer serves as a bottom gate (BG). The length of the device is approximated in terms of the corresponding moiré wavelength (*λ*_m_). (b) Two-probe conductance *G* as a function of filling factor *ν* for three different devices measured across top and bottom contacts (configuration-TB; *T* = 0.26 K). The inset in the bottom panel shows the zoomed in region near the full fillings of the band (*ν* = ±4; marked by dashed rectangular regions). (c) Schematic showing the tunneling process in three different regimes. For the large device, the electron diffuses from drain to source contact, whereas for the small area devices, the electrons tunnel from drain to a local quantum dot and then to the bulk of the sample before finally moving to source contact. The quantum dot is defined by a local potential well and the confined states within this potential well are indicated by horizontal lines.

The graphene and hexagonal boron nitride (h-BN) flakes were obtained *via* mechanical exfoliation of the respective crystals, and the TBLG devices were fabricated using the tear-and-stack dry transfer method^25,26^ (see Methods for the detailed fabrication process). The layer thickness was identified by optical microscopy and confirmed by Raman spectroscopy prior to device fabrication. In order to probe the effect of dimensionality and confinement on the transport properties, three TBLG devices were fabricated: device S1 (*θ* ≈ 1.65°) had a large area (∼2.8 × 1.7 μm), while S2 (*θ* ≈ 1.6°) and S3 (*θ* ≈ 1.19°) were confined to smaller rectangular geometries (∼500 × 250 nm). Fig. 1a presents schematic illustrations of our h-BN encapsulated TBLG devices with varying device dimensions and twist angles. The TBLG is contacted through 1D edge contacts with Cr/Au electrodes, with graphite serving as a local back gate. The devices were patterned with contacts on all four sides (Fig. 1a) to probe different configurations within the same device.

Fig. 1b shows the two-probe conductance *G* measured between top and bottom contacts (TB configuration) as a function of the band filling fraction *ν* (*ν* = *n*/*n*_0_, where *n*_0_ is the density of one electron/hole in the unit cell) in three different devices at *T* = 0.26 K. The recurring features in *G* across the tBLG devices can be identified as minima in *G* at the charge neutrality point (CNP) and at the full-filling of the lowest moiré bands (*ν* = ±4).^3^ In addition, device S3 exhibits minima in *G* close to half filling of the bands (*ν* = ±2), which is expected to result from the strong electronic correlations close to the magic angle.^3^ Furthermore, for devices S2 and S3, the conductance exhibits notably more features in the form of reproducible oscillations as the density is tuned across the bands. Remarkably, this effect is most pronounced in device S3, and is closest to *θ*_m_. As the Fermi energy (*E*_F_) is tuned to the edge of flat bands (*ν* ≥ ±4) device S3 exhibits non-periodic reproducible conductance oscillations, as shown in the inset of the bottom panel in Fig. 1b.

**Fig. 2:**
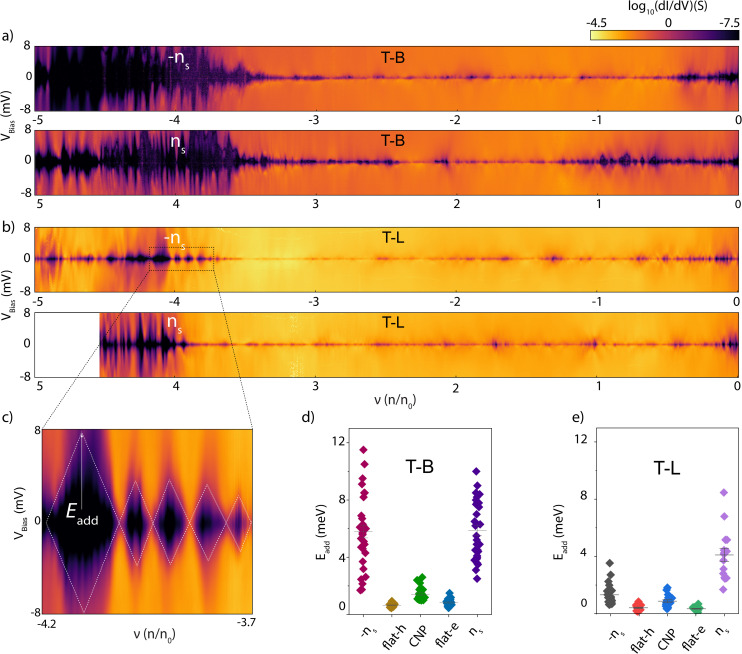
Differential conductance and quantum dot behaviour. (a) and (b) Differential conductance d*I*/d*V* of the device S3 as a function of *V*_bias_ and *ν* for two different configurations. The device exhibits diamond like structures, clearly pronounced at the edge of the flat bands. (c) Zoomed-in view near the band filling of *ν* = −4, for the TL configuration, showing the closing diamonds. (d) and (e) Addition energy *E*_add_ extracted using the Coulomb diamonds in (a) and (b), respectively. Flat-h and flat-e correspond to the intermediate density regions in the flat bands (0 < |*ν*| < 4).

To understand the origin of the observed conductance oscillations, we measured the differential conductance (d*I*/d*V*) as a function of source–drain bias *V*_bias_ and *ν* for different contact configurations in device S3, two of which are shown in Fig. 2a and b. The conductance maps recorded on the other configurations and device S2 are presented in Fig. 1 and 2 of the ESI.† Near the edges of the flat bands, the plots reveal irregular and aperiodic diamond-shaped patterns. A few fainter diamonds appear in other density regions as well. These diamond-shaped regions (black areas) represent Coulomb diamonds (CDs). CDs show signature quantum dot (QD) behavior which arises due to a combination of quantum confinement of the charge carriers and coulombic interactions between charge carriers. Within each CD, a specific, fixed number of electrons occupies the QD. Charge transport occurs only when the electrochemical potential of the QD aligns with the bias window, which occurs along the diamond edges. Tracking the crossing points of diamond edges as a function of charge carrier density provides spectroscopic insight into the electronic levels. The height of each CD corresponds to the addition energy, which is the energy required to add or remove a single electron from the QD. From this observation, we deduce that the conductance peaks in Fig. 1 are Coulomb blockade (CB) oscillations. We note that while CB resonances become more pronounced in device S3 (closest to *θ*_m_), their appearance is also influenced by factors such as the strength of the insulating states (*e.g.*, the band gap size) and local doping variations. Our data indicate that the device with the twist angle closest to the magic angle exhibits the largest band gap among the measured devices, which may contribute to the observed behavior. This suggests that while the twist angle plays a significant role, the relationship between resonance behavior and the magic angle is complex and may not be straightforward.

For a large range of charge carrier densities, band transport occurs, and the sample is in a conducting state. While CDs emerge around the edge of the flat bands (*ν* = ±4), many do not show observable crossings of the corresponding energy level with the Fermi energy of the electrodes. We only observed closing CDs at specific filling fractions and for specific contact configurations, for example near *ν* = −4 (Fig. 2c) in the TL configuration, a situation corresponding to the charge transport through a single QD. Overall, these observations point to a device active area composed of multiple conducting regions isolated from each other by insulating regions. The conducting regions behave as local QDs with discrete energy levels, whereas the non-conducting areas act as a tunnel barrier between the conducting regions. Depending on the contact configuration and charge carrier density, we observed situations where a single QD contributes to transport, as well as situations where several weakly coupled QDs, either in series or potentially in parallel, contribute to transport.^27^ We depict this scenario with a tunneling diagram in Fig. 1c. For readability, a single QD connected *via* bulk electrodes is presented.

**Fig. 3:**
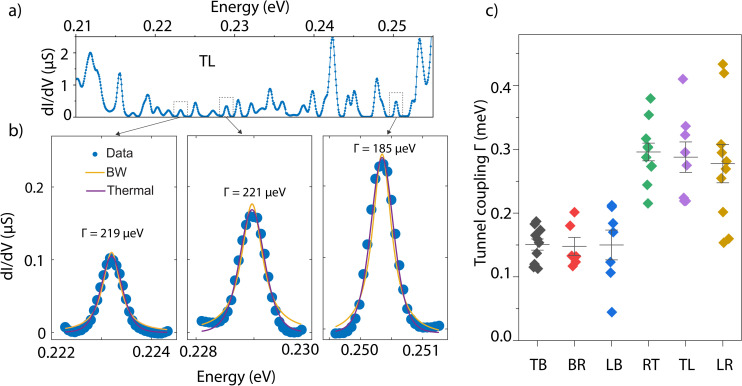
Tunnel coupling fitting for Coulomb resonances. (a) d*I*/d*V* in a selected range of energy near the band edge (*ν* = 4) for device S3. The band filling is converted to energy by estimating the gate coupling from Coulomb diamonds. (b) CB resonance nonlinear fittings for three representative conductance peaks (marked in a). The solid yellow curve is the Breit–Wigner (BW) model fitting, whereas the solid magenta curve is pure thermally broadened resonance fitting. (c) Extracted coupling for various CB resonances observed in different measurement configurations.

The CD data in Fig. 2d and e are useful in gaining deeper insight into the QD geometry. For a closing diamond with an addition energy of ∼2 meV, we can estimate a QD size of about 525 nm, using a disc model.^28^ This aligns well with the device size of 430 nm. For the maximum observed addition energy of ∼12 meV, the estimated size of the QD is ∼90 nm. These observations suggest that depending on the charge carrier density, the charges are localized in different regions of the device. These findings are consistent with previous measurements. STM studies in similar devices have reported typical QD sizes around 60 nm,^24^ while tunneling measurements have shown QD sizes up to 600 nm. This range supports our observations of varying QD sizes within the device. From the measured CDs, we also extracted the gate coupling and converted the gate voltage axis into energy. Using the Breit–Wigner (BW) model^29^ for resonant transport through a single-lifetime-broadened transport level, we then determined the total tunnel coupling *Γ* of the QDs to the leads. Fig. 3b shows the BW model (yellow curve) fitted to three representative conductance peaks, yielding extracted couplings ranging from ∼185 to 221 μeV. To ascertain that the resonances are not solely temperature-broadened, we fit our data to thermally broadened resonances (magenta curve). The analysis reveals an equivalent temperature of approximately 2–3 K, which is an order of magnitude higher than the cryostat temperature (0.26 K), indicating that the broadening of the resonances observed in our measurements is primarily due to the hybridization of electronic levels with the electrodes. Fig. 3c shows a comparison of the tunnel couplings extracted across different configurations in the same device. We note that the coupling strength is influenced by the bandgap and physical structure of the insulating regions, which vary with the configuration, gate voltage, and the strength of local doping inhomogeneity, leading to significant variations in tunnel coupling strength.

**Fig. 4:**
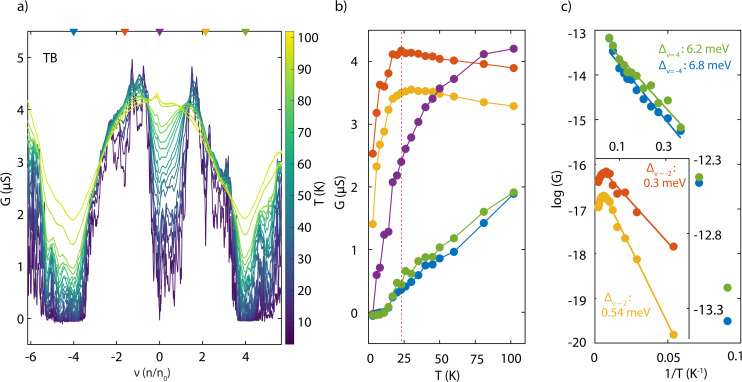
Temperature dependent charge transport. (a) Conductance *G* of the device S3 as a function of the filling factor *ν* at different temperatures ranging from 0.26 K to 105 K. (b) *G* as a function of *T* at five different values of *ν* (marked in panel a, top axis). The dashed line show a transition from metallic to insulating behavior at conductance dips near *ν* = ±2. (c) Arrhenius plots of *G* at full fillings of the superlattice bands (*ν* = ±4). The solid lines are Arrhenius fits to the data in the temperature regime above 15–20 K, where *Δ* is the extracted activation energy of the device. The inset shows similar fits to the conductance minima observed near half-fillings of the bands (*ν* ∼ ±2).

To gain further insights into the energetics of the system, we investigated the temperature dependence of the conductance in three different configurations. Fig. 4a shows conductance *G* as a function of band filling *ν*, measured for the TB configuration (see ESI Fig. 4† for two other configurations) in a temperature range of 0.26–105 K. Conductance *G vs*. temperature *T* traces are shown in Fig. 4b for the selected fillings indicated on panel a (top axis). We observed an insulating behaviour at the CNP and *ν* = ±4 in the measured range of temperature. At intermediate fillings (orange and yellow traces), we found an insulating behaviour at low *T* (∼0.26–25 K), followed by a metallic behaviour as the temperature increased further.

To estimate the intrinsic activation gap, an Arrhenius plot of the temperature dependence of *G* at *ν* = ±4 is shown in Fig. 4c. The experimental data above 15–20 K follow the expected behaviour for thermally activated transport, according to *G* = *G*_0_ exp(−*Δ*/2*k*_B_*T*), where *k*_B_ is the Boltzmann constant and *G*_0_ and *Δ* are fitting parameters. Activation gaps of 6.2 meV and 6.8 meV are extracted for *ν* = ±4, respectively. At lower values of *T*, the deviation from the thermally activated behaviour to a much weaker *T*-dependence can be attributed to a combination of the Mott variable-range hopping conduction mediated by localized states and quantum tunnelling through a short-channel semiconductor.^30^ Additionally, the conductance minima close to *ν* = ±2 also showed an activated type behaviour at low *T* (<20 K), although with a much smaller activation gap of 0.54 meV and 0.3 meV, respectively (inset of Fig. 4c). We compared the activation energy at full filling across three different configurations for the same device (see Fig. 5, ESI†). We note that, although the twist angles measured for different configurations are almost similar (within 0.01°, as measured by electric transport data), there is a large variation in the activation energies, thereby emphasizing the role of doping inhomogeneity in the device. Our findings provide insight into the varying activation gaps observed across devices in previous studies, highlighting the impact of spatial doping variations.^3,11,26,31^

We now discuss the possible reasons behind the observed quantum dot physics. Coulomb oscillations indicate the presence of local confinement potentials, which can arise from variations in the twist angle across the sample geometry,^22^ spatial doping variations within the sample,^24^ or edge defects. Indeed, a recent study has shown that local twist angle variations may result in CB effects.^32^ However, the combination of almost identical twist angles, significant variations in activation gaps, and the splitting of the conductance dip at the CNP at low temperatures suggests that spatial doping variations may be a more plausible scenario. Under the influence of these local doping variations, certain domains may become insulating earlier than others, leading to QD behavior near the band edge in our devices. However, as the devices are lithographically defined, one cannot exclude that the QD behavior originates from defect states that are localized along the edges of the devices. One might also anticipate observing CDs near half-filled insulators. However, while we do observe some weak non-closing diamonds in the flat band region, a clear signature of confinement near half-filled states is absent. This is possibly due to the very small energy gaps at half-filling regimes. Alternatively, it could be due to a stronger screening of the disorder potential by the higher density of states, leading to a weaker confinement.^24^

Despite the encapsulation of TBLG with h-BN and the use of graphite as a back gate to suppress the effects of charge impurities and potential disorder, our observations show that spatial doping variations may still be present, as also previously observed in STM measurements.^24^ Doping variations can for example arise from substrate-induced inhomogeneity, defects in the h-BN, and trapped water or organic impurities during heterostructure fabrication. Moreover, we observed the splitting of the conductance dip at the CNP in other devices as well, emphasizing the role of doping variations across devices (see Fig. 3, ESI†). We believe that, owing to the flat electronic bands and the energy gaps at integer fillings, even the slightest doping variation is amplified in TBLG, leading to significant alterations in the overall transport properties of the system.^24^ This effect has so far been elusive in transport experiments, where the device size is usually much larger than the size of QDs formed in the channel. Further experiments involving gate-defined QDs in TBLG will be pivotal for understanding the transport properties at quantum length scales while minimizing edge disorder.

## III. Conclusion

In summary, we investigated the doping inhomogeneity in twisted bilayer graphene through charge transport measurements, utilizing various device dimensions and twist angles, which led to the emergence of quantum dot behavior. Our measurements suggest that charge carriers are confined in conducting regions separated by insulating regions, forming Coulomb diamonds near the edge of flat bands. We observed that, even when the device size is reduced to less than 0.5 μm and the twist angles measured for different configurations are similar, there is a significant variation in the activation gaps, highlighting the role of spatial doping variations in the sample. Our findings suggest that magic-angle twisted bilayer graphene (TBLG) samples are highly sensitive to electrostatically weak random disorder potentials, providing insights into the observed substantial variations across the samples, particularly in their electrical transport properties.

## IV. Methods

### Device fabrication

The TBLG devices were fabricated following a typical ‘tear and stack’ technique.^25,26^ Graphene, hBN (35–45 nm and 3–6 nm thick flakes for bottom and top layers, respectively) and graphite flakes were mechanically exfoliated on SiO_2_/Si (100 nm oxide thickness). The thickness of h-BN layers was determined by using AFM in the tapping mode (Bruker Icon3 AFM). Subsequently, the heterostructure was assembled employing dry-transfer techniques.^33^ Initially, poly bisphenol A carbonate (PC) supported by polydimethylsiloxane (PDMS) on a glass slide was utilized to pick up the h-BN layer, followed by tearing and picking up the graphene flake. A commercial micromanipulator from HQ graphene facilitated control over the rotation angle. Finally, the graphite flake was picked up to serve as a bottom gate. The fabrication of electrodes followed a standard electron-beam lithography process and electron-beam metal evaporation. The devices were structured into a four-terminal configuration using reactive ion etching with a CHF_3_ and O_2_ gas mixture. All contacts were patterned as 1D edge contacts with Cr/Au electrodes.^33^

### Electronic measurements

All electronic measurements were performed under vacuum conditions (<10^−6^ mbar). Transport measurements were carried out in a He_3_ cryostat with a base temperature of 255 mK. For DC measurements, a data acquisition board (ADwin-Gold II, Jäger Computergesteuerte Messtechnik GmbH) was employed to apply bias and gate voltages. The voltage output was then measured using an I–V converter (DDPCA-300, FEMTO Messtechnik GmbH).

### Twist angle estimation

To estimate the twist angle, we utilized the relationship 
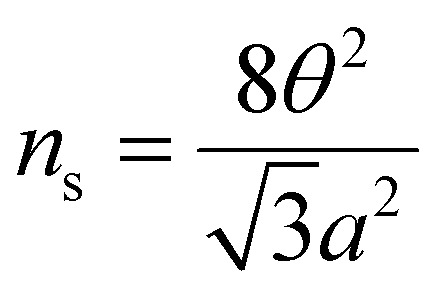
, which accounts for both valley and spin degeneracies. Here, *a* = 0.246 nm represents the lattice constant of graphene, and *n*_s_ denotes the charge carrier density corresponding to a fully filled superlattice unit cell.^3^ The conductance minima observed at a slightly elevated temperature (∼10 K) were used to determine the value of *n*_s_, as the conductance remained flat over an extended range of density at the lowest temperature (∼0.26 K).

## Author contributions

J. Z. conceived and designed the experiments. P. B. fabricated the devices. J. Z., B. G., P. B., and M. L. P. performed the electrical measurements. K. W. and T. T. provided h-BN crystals. B. G., J. Z. and M. L. P. analyzed the data. B. G., J. Z., M. L. P., and M. C. discussed the figures and wrote the manuscript. All authors discussed the results and their implications and commented on the manuscript.

## Data availability

The data supporting this article have been included as part of the ESI.†

## Conflicts of interest

The authors declare that there are no competing interests.

## Supplementary Material

NR-017-D4NR02824D-s001
