# Supplementary material for: Electronic confinement induced quantum dot behavior in magic-angle twisted bilayer graphene
Source: Nanoscale. 2024 Dec 23;17(7):4030–7. doi: 10.1039/d4nr02824d (PMC11705233; doi:10.1039/d4nr02824d)
Supplement: NR-017-D4NR02824D-s001 [file NR-017-D4NR02824D-s001.pdf]

Supplementary Information (SI) for Nanoscale.
This journal is © The Royal Society of Chemistry 2024

Supplementary information: Electronic confinement induced quantum dot behavior in magic-angle twisted bilayer graphene

Bhaskar Ghawri,[1] Pablo Bastante,[2] Kenji Watanabe,[3] Takashi Taniguchi,[4] Michel Calame*,[1,5,6] Mickael L. Perrin*,[1,7,8] and Jian Zhang*[1,9]

[1] *Transport at Nanoscale Interfaces Laboratory, Empa, Swiss Federal Laboratories for Materials Science and Technology, 8600 Dübendorf, Switzerland*

[2] *Departamento de Física de la Materia Condensada, Universidad Autónoma de Madrid, 28049, Madrid, Spain*

[3] *Research Center for Functional Materials, National Institute for Materials Science, 1-1 Namiki, Tsukuba 305-0044, Japan*

[4] *International Center for Materials Nanoarchitectonics, National Institute for Materials Science, 1-1 Namiki, Tsukuba 305-0044, Japan*

[5] *Department of Physics, University of Basel, 4056 Basel, Switzerland*

[6] *Swiss Nanoscience Institute, University of Basel, 4056 Basel, Switzerland*

[7] *Department of Information Technology and Electrical Engineering, ETH Zurich, 8092 Zurich, Switzerland*

[8] *Quantum Center, ETH Zürich, 8093 Zürich, Switzerland*

[9] *Max Planck Institute of Microstructure Physics, Weinberg 2, Halle 06120, Germany*

*$mickael.perrin@empa.ch; michel.calame@empa.ch; jian.zhang@mpi-halle.mpg.de$

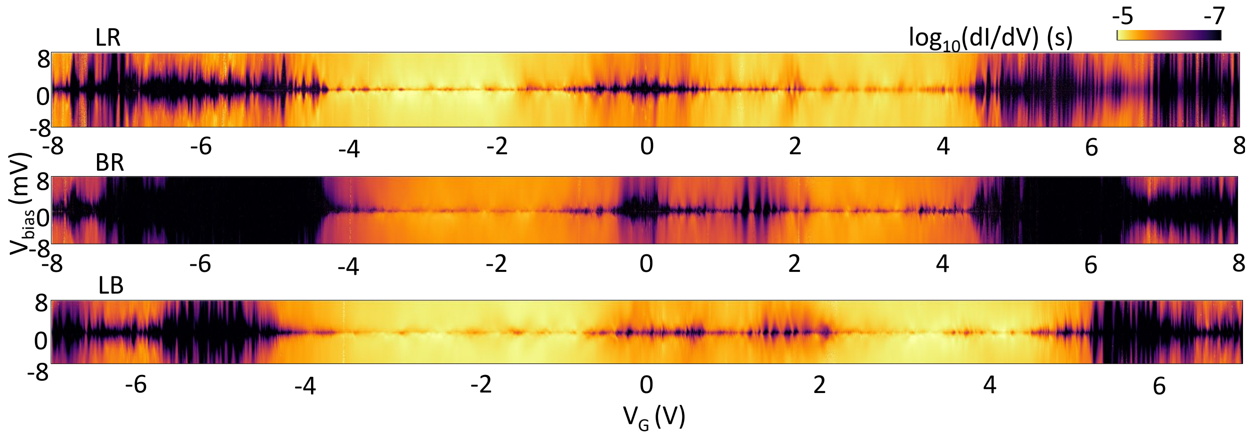

Supplementary Figure 1: **Differential conductance and quantum dot behaviour in device S3.** Differential conductance dI/dV of the device S3 as function of V_{Bias} and V_G for three different configurations, which are not presented in main manuscript. Similar to the other two configurations shown in main text, the device exhibits diamond like structures, clearly pronounced at the edge of the flat bands. Although all configurations exhibit Coulomb diamonds, the exact shape and addition energies of diamonds vary across configurations, thereby highlighting the role of doping inhomogeneity across the device.

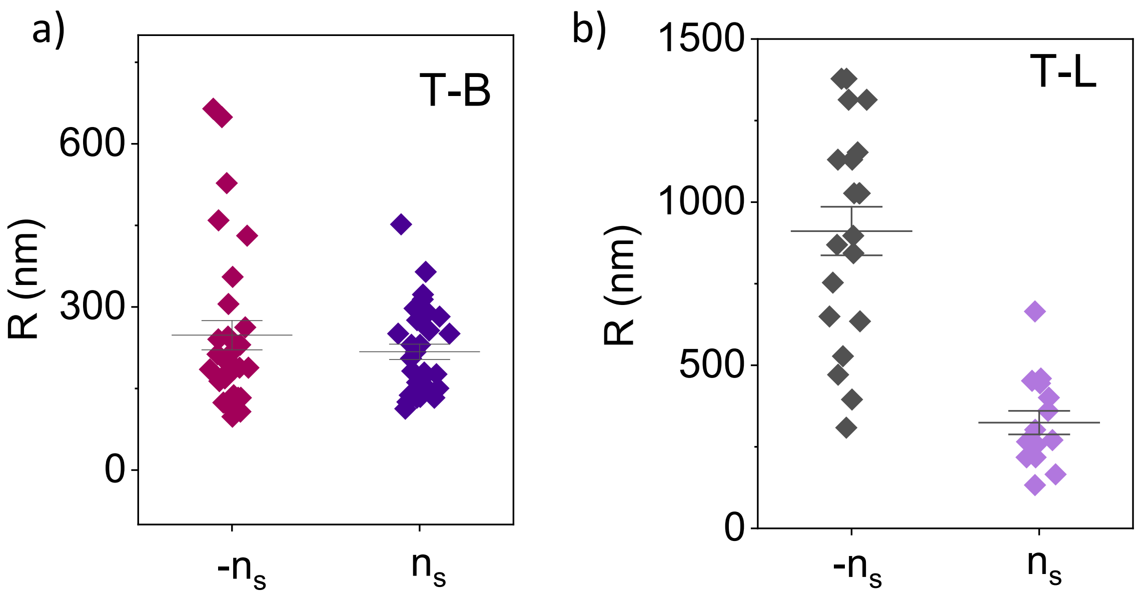

Supplementary Figure 2: **Estimated size of quantum dot behaviour in device S3. a), b)** Upper bound on quantum dot size using Coulomb diamonds data and a circular disc model in configuration T-B and T-L, respectively. Only diamonds near the edge of flat bands are used for the calculation, as diamonds within the flat bands are not pronounced. It is important to note that the size and exact physical structure of these quantum dots varies with changes in gate voltage. For very small diamonds, such as those near $-n_s$ in Fig. 2b, the addition energies are very small, which can make size calculations unreliable; in some cases, they may even exceed the physical size of the device (see Fig. **b)**).

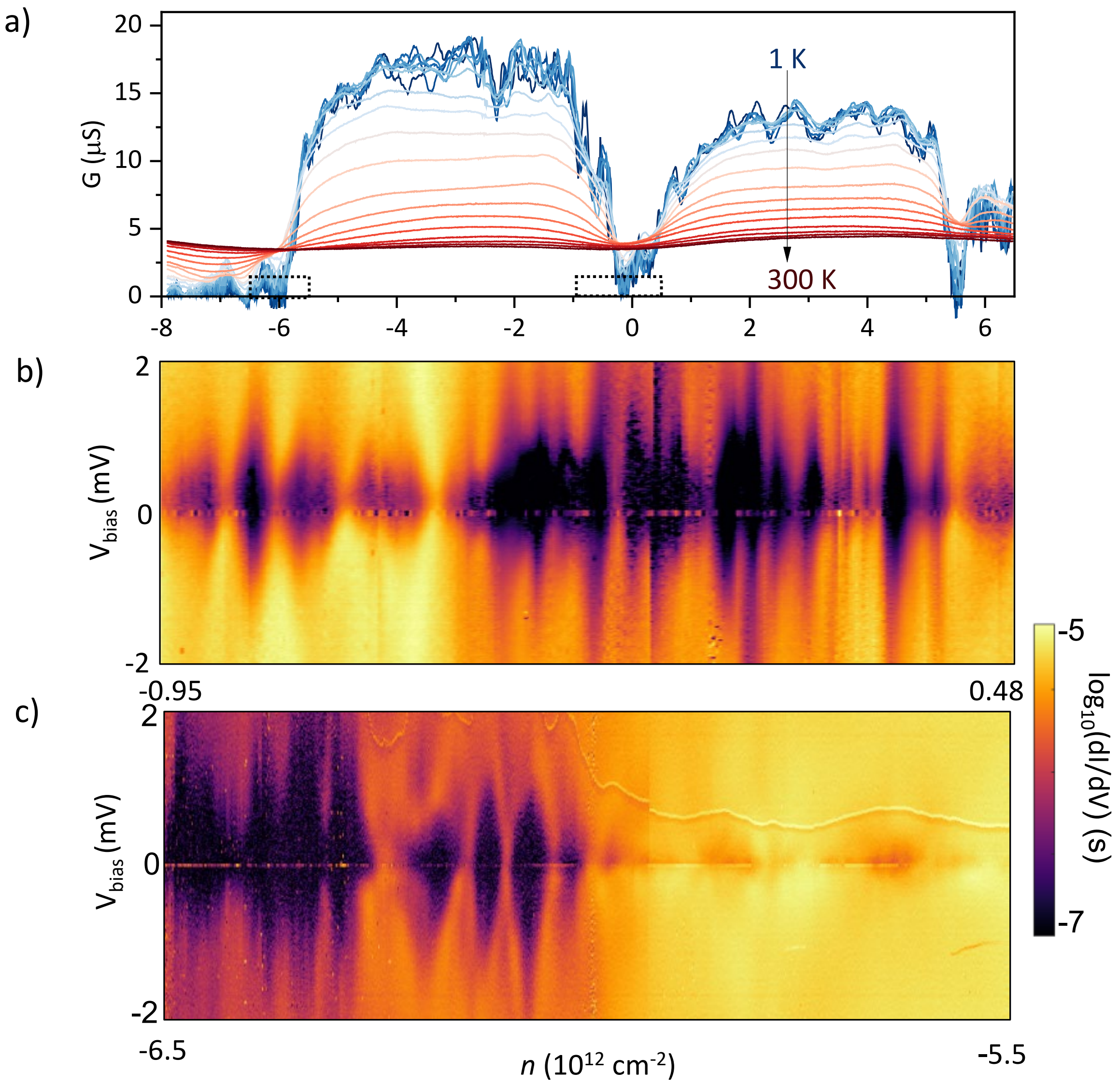

Supplementary Figure 3: **Charge transport in device S2. a)** Conductance G of the device S2 as function of number density n at different temperatures ranging from 1 K to 300 K in configuration T-B. Similar to device S3 (Fig. 4 in main text), the splitting of the conductance dip at the CNP is observed in this device as well. **b), c)** Differential conductance dI/dV as function of V_{Bias} and ν for two different density regions near CNP and $\nu = -4$ (shown by dashed rectangles in **a)**), respectively.

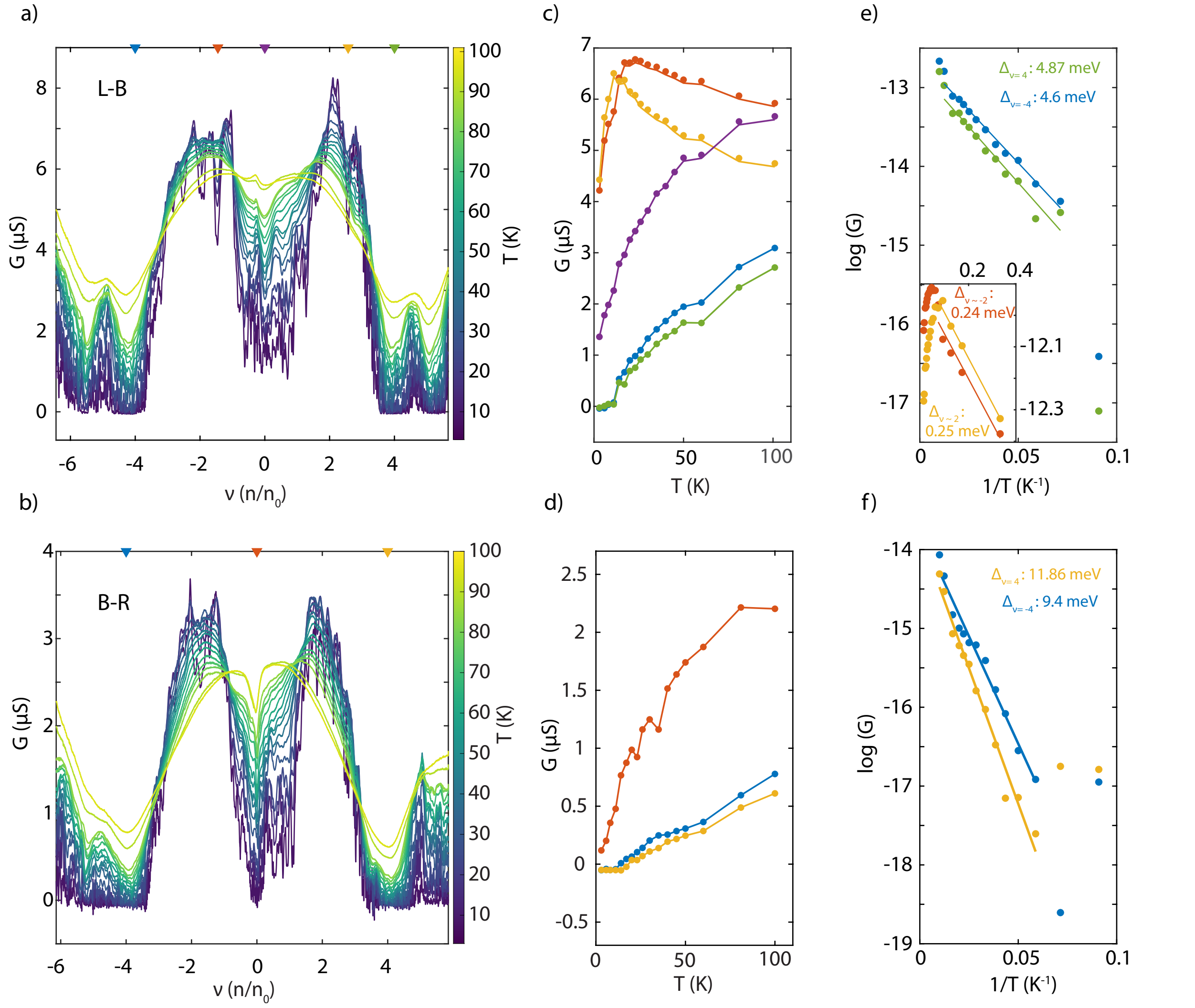

Supplementary Figure 4: **Charge transport in different configurations in device S3**. **a), b)** Conductance G of the device S3 as a function of filling factor ν at different temperatures ranging from 3 K to 100 K in configuration L-B and B-R, respectively. **c)** G as a function of T at five different values of ν (marked in panel a, top axis) in configuration L-B. Similar to configuration T-B shown in main manuscript, we observe an insulating behaviour at the CNP and $\nu = \pm 4$ in the measured range of temperature. At intermediate fillings (orange and yellow traces), we find an insulating behaviour at low-T ($T < 15 - 20$ K), followed by a metallic behaviour as temperature increases further. **d)** G as a function of T at three different values of ν (marked in panel b, top axis) in configuration DR. This configuration does not show pronounced conductance dips near ($\nu \sim \pm 2$). **e), f)** Arrhenius plots of G at full fillings of the superlattice bands ($\nu = \pm 4$) for configuration L-B and B-R, respectively. The solid lines are Arrhenius fits to the data in the temperature regime above $15 - 20$ K, where Δ is the extracted activation energy of the device. The inset in **e)** shows similar fits to the conductance minima observed near half-fillings of the bands ($\nu \sim \pm 2$)).

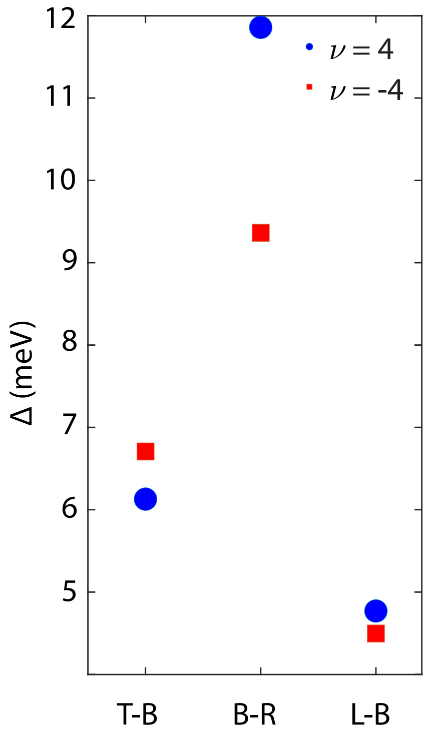

Supplementary Figure 5: **Activation energies in device S3.** Measured activation energy Δ at $\nu = \pm 4$ for three different configurations in device S3. We observe that, despite the twist angles being nearly identical across different configurations (within 0.01° as determined by electric transport data), there is significant variation in the activation energies. This highlights the impact of doping inhomogeneity in the device.
